# Supplementary figures and images for: Phenotype stability and dynamics of transposable elements in a strain of the microalga Tisochrysis lutea with improved lipid traits
Source: PLoS One. 2023 Apr 27;18(4):e0284656. doi: 10.1371/journal.pone.0284656 (PMC10138859; doi:10.1371/journal.pone.0284656)

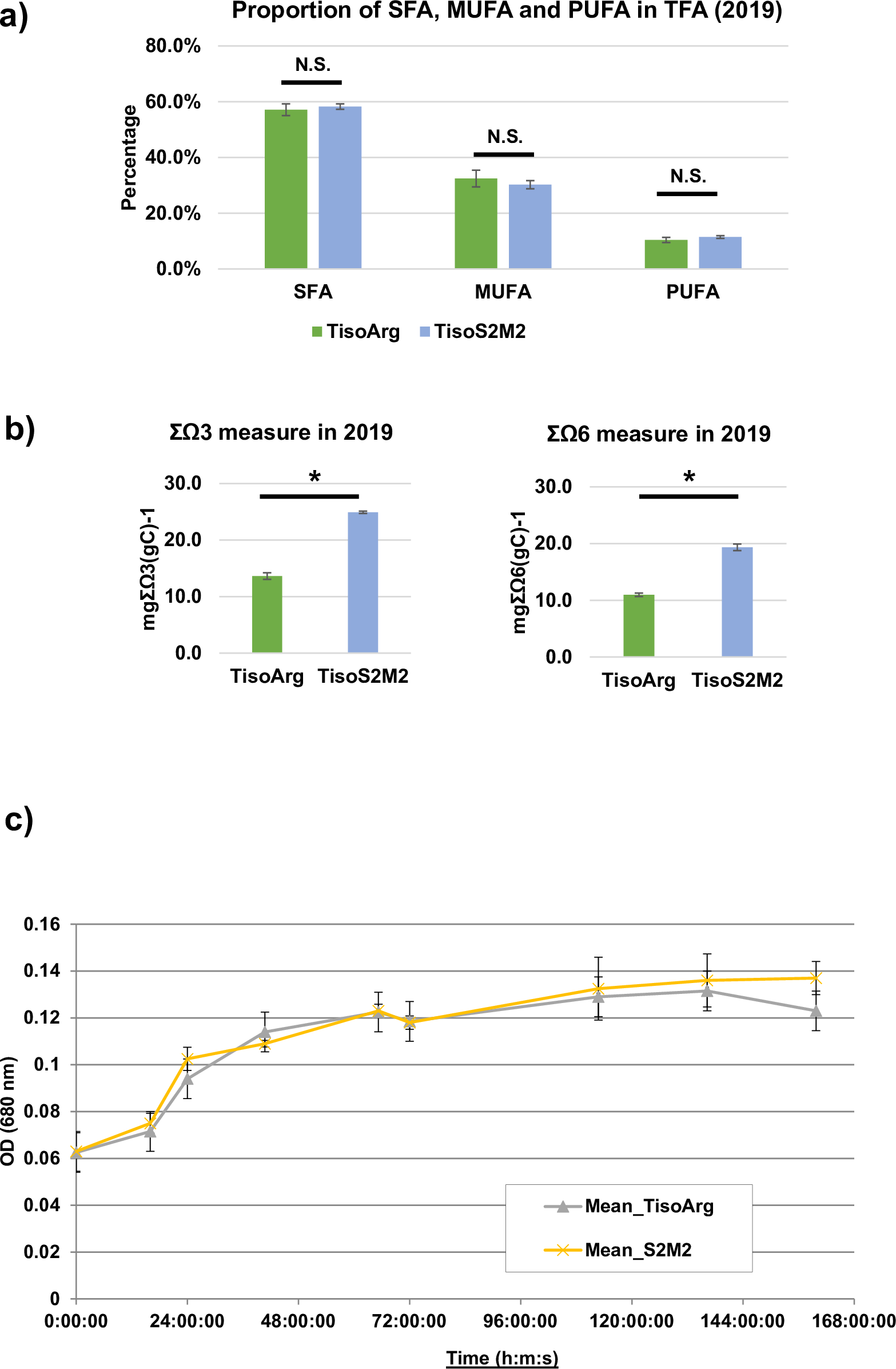

Supplement: S1 Fig — a) Comparison of the proportion of SFA, MUFA and PUFA. b) Comparison of the ƩΩ3 and ƩΩ6 content. c) Growth curves of TisoArg and TisoS2M2. For a) and b), bars represent the means of two biological replicates ± SD. *, p < 0.05 by t-test. N.S. means Not significant. For c) Each dot represents the mean of two biological replicates ± SD. (TIF) [file pone.0284656.s001.tif]

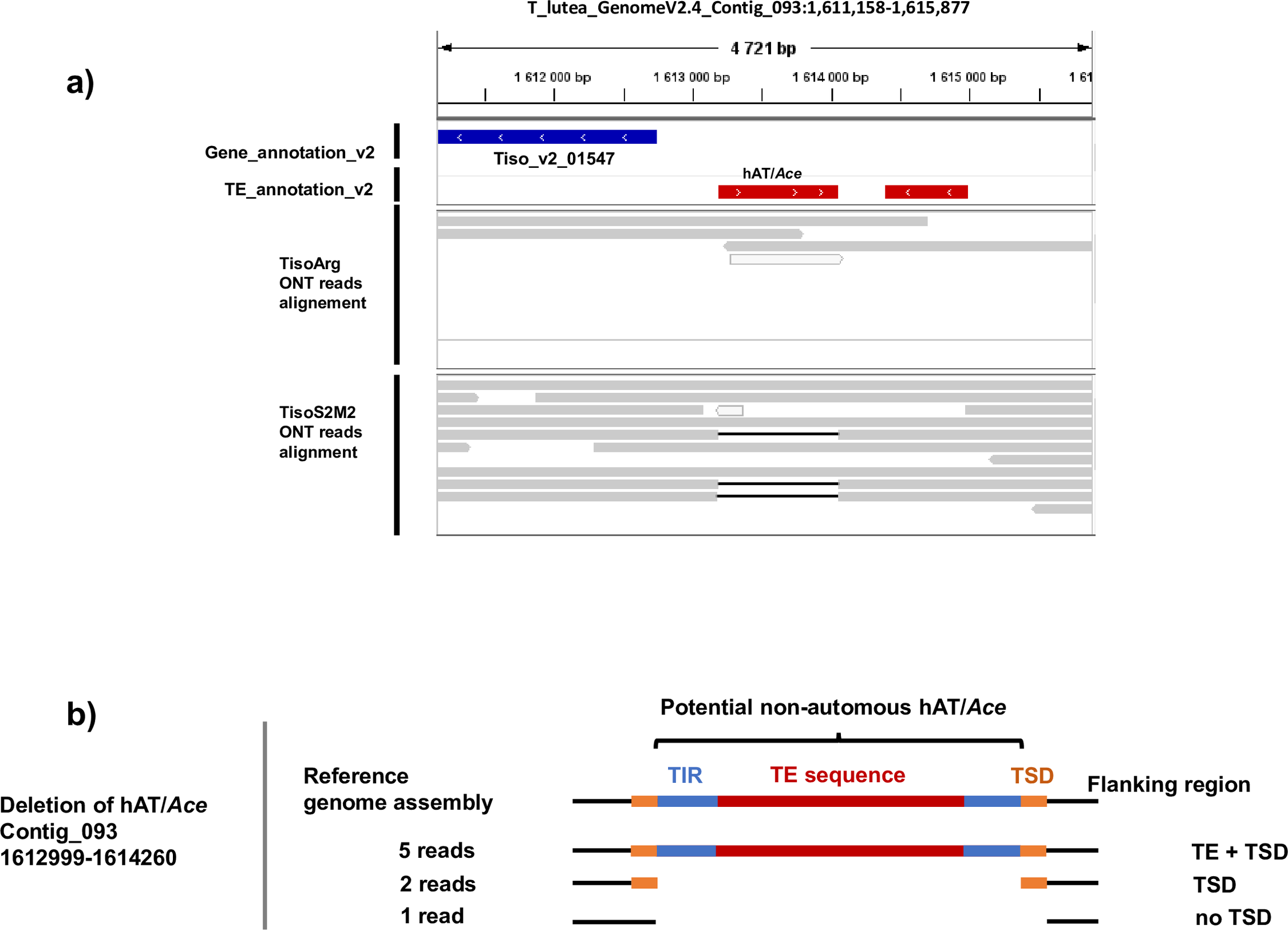

Supplement: S2 Fig — a) Track of the locus at Contig 93 with a predicted hAT/Ace deletion in TisoS2M2. The TE deletion event is visible for 3 reads. b) Illustration of the heterozygosity at the loci of the hAT/Ace deletion events at Contig 93 and the supporting nucleic motifs found from the long reads of TisoS2M2. The highlight of the motifs are in S7 Data. (TIF) [file pone.0284656.s002.tif]

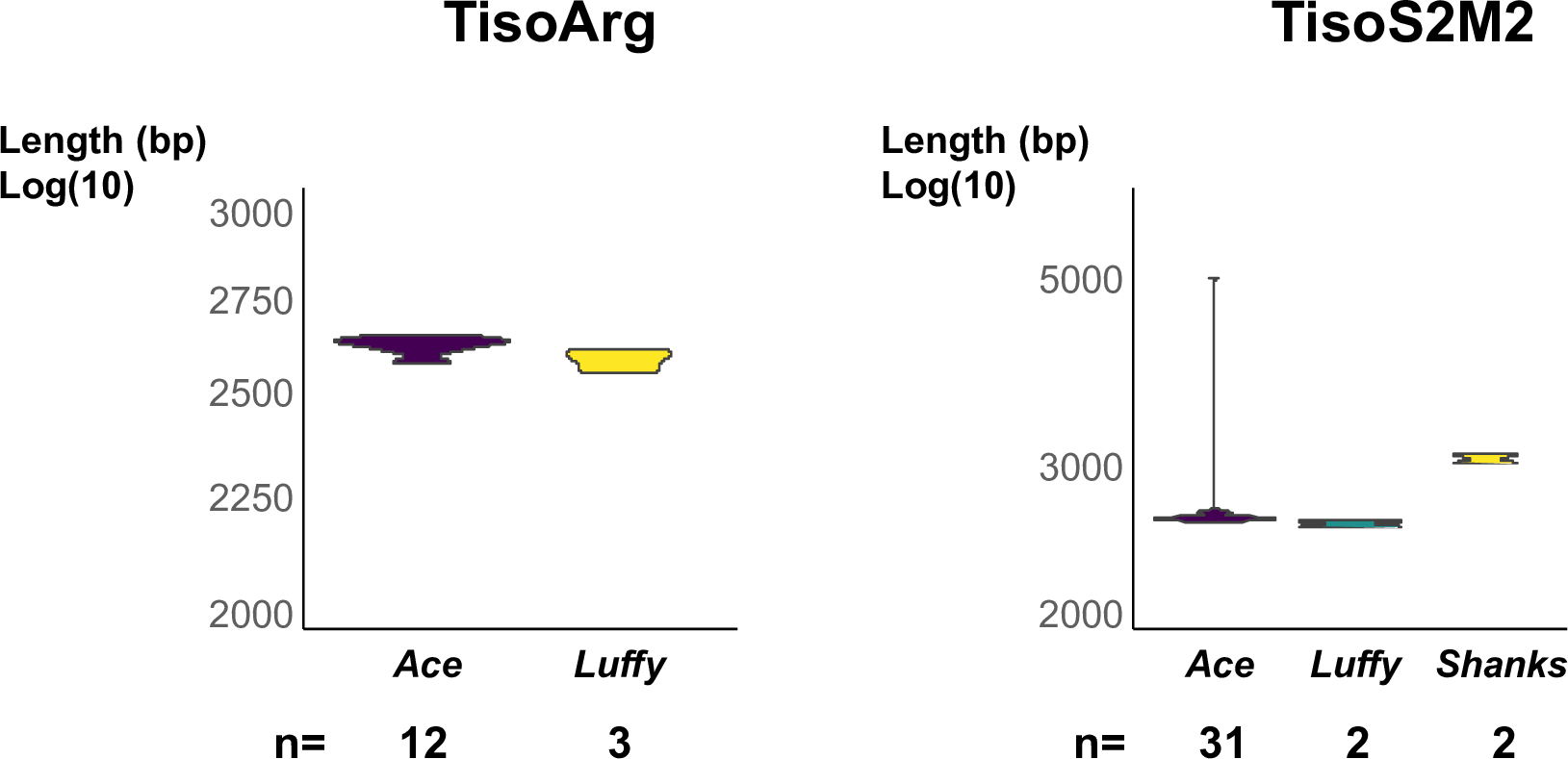

Supplement: S3 Fig — Lengths were retrieved from Sniffles predictions in S4 Data). TE families with one predicted insertion are not shown. (TIF) [file pone.0284656.s003.tif]

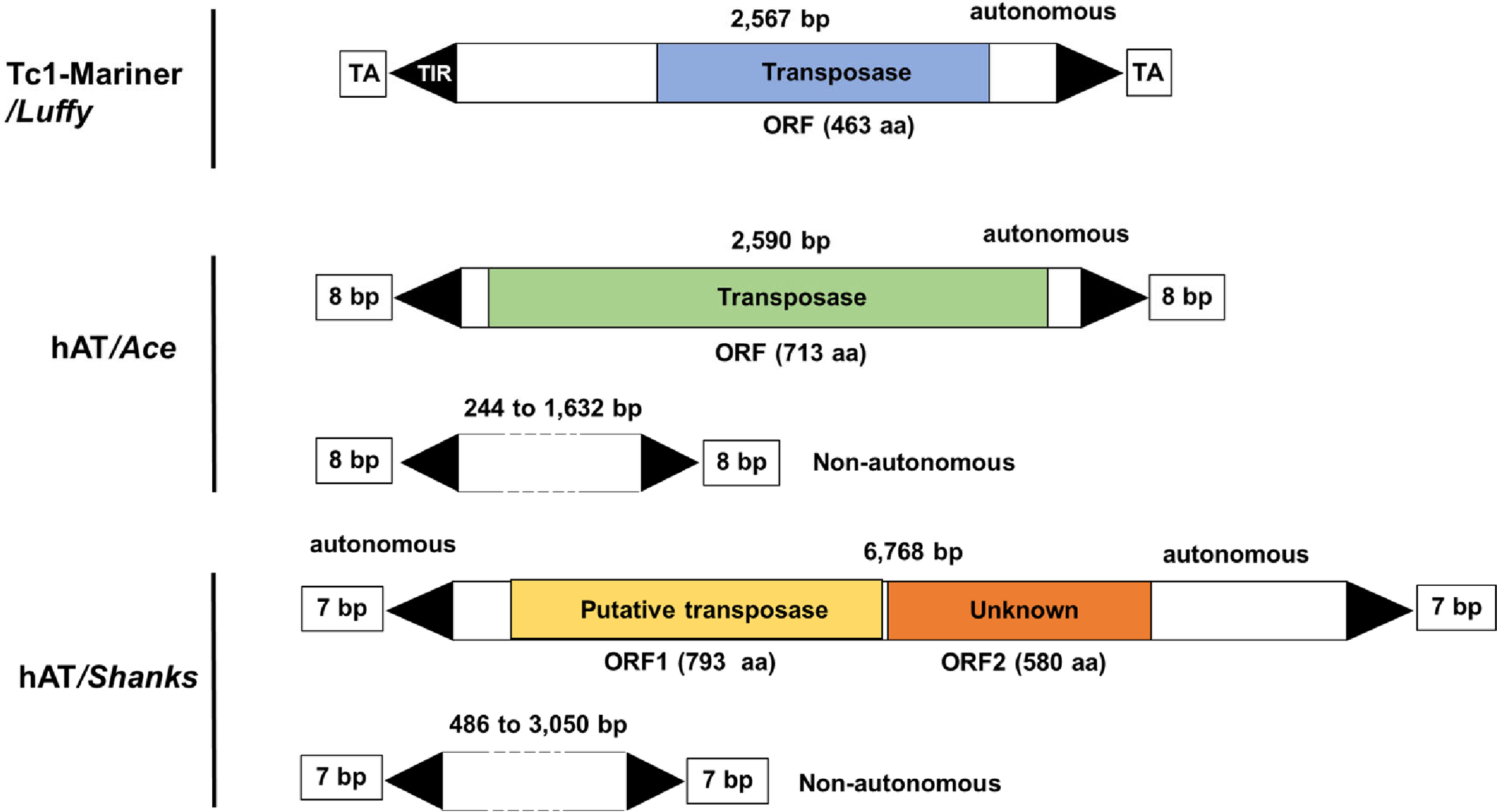

Supplement: S4 Fig — (TIF) [file pone.0284656.s004.tif]

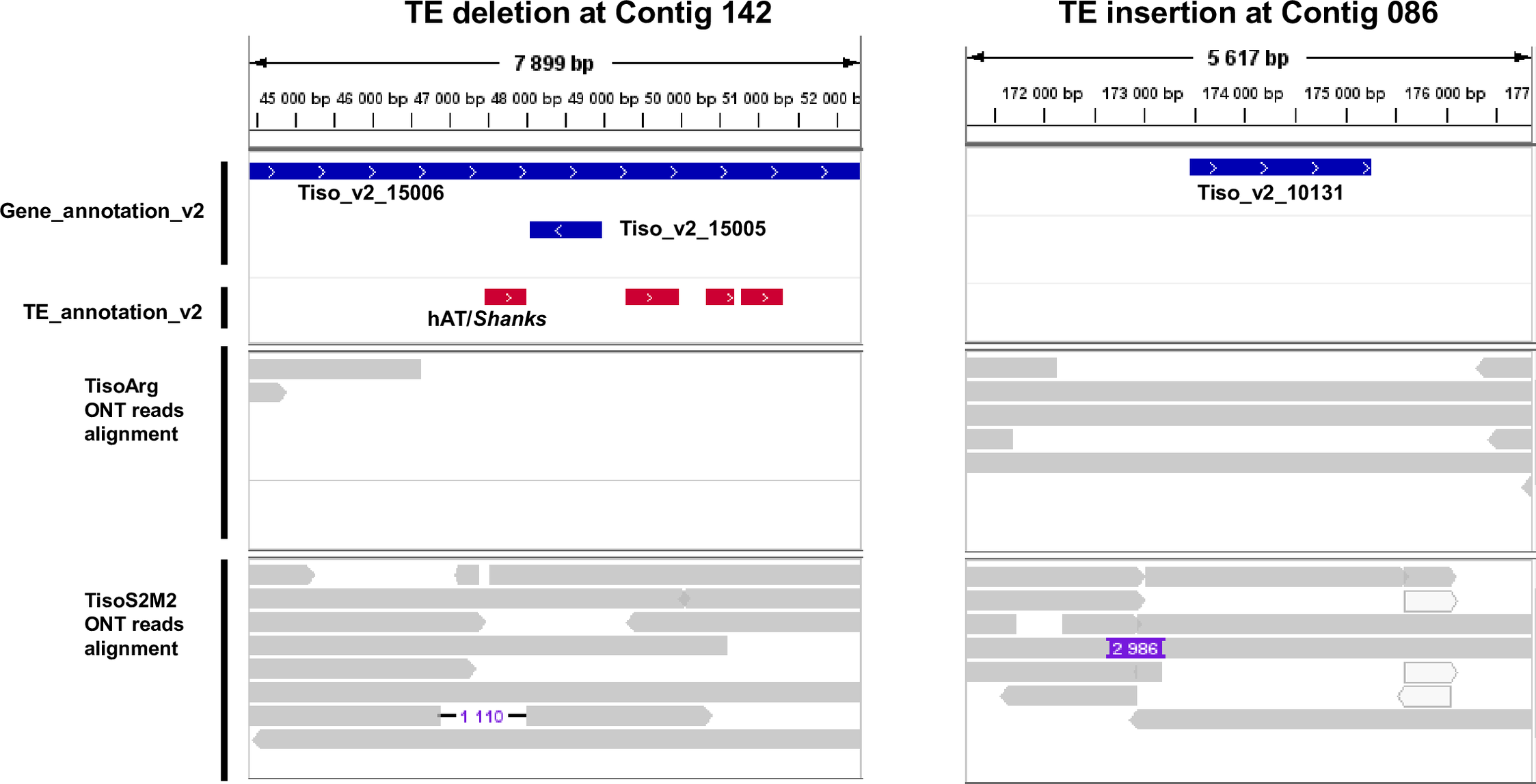

Supplement: S5 Fig — a) A TE deletion is predicted close to a gene encoding for a class 3 lipase in TisoS2M2. b) A TE insertion is predicted to be closely located to a gene encoding for a CDK. The indels are highlighted in violet and the lengths of the events are indicated. (TIF) [file pone.0284656.s005.tif]
