# Supplementary material for: Phenotype stability and dynamics of transposable elements in a strain of the microalga Tisochrysis lutea with improved lipid traits
Source: PLoS One. 2023 Apr 27;18(4):e0284656. doi: 10.1371/journal.pone.0284656 (PMC10138859; doi:10.1371/journal.pone.0284656)
Supplement: S3 Data — (PDF) [file pone.0284656.s008.pdf]

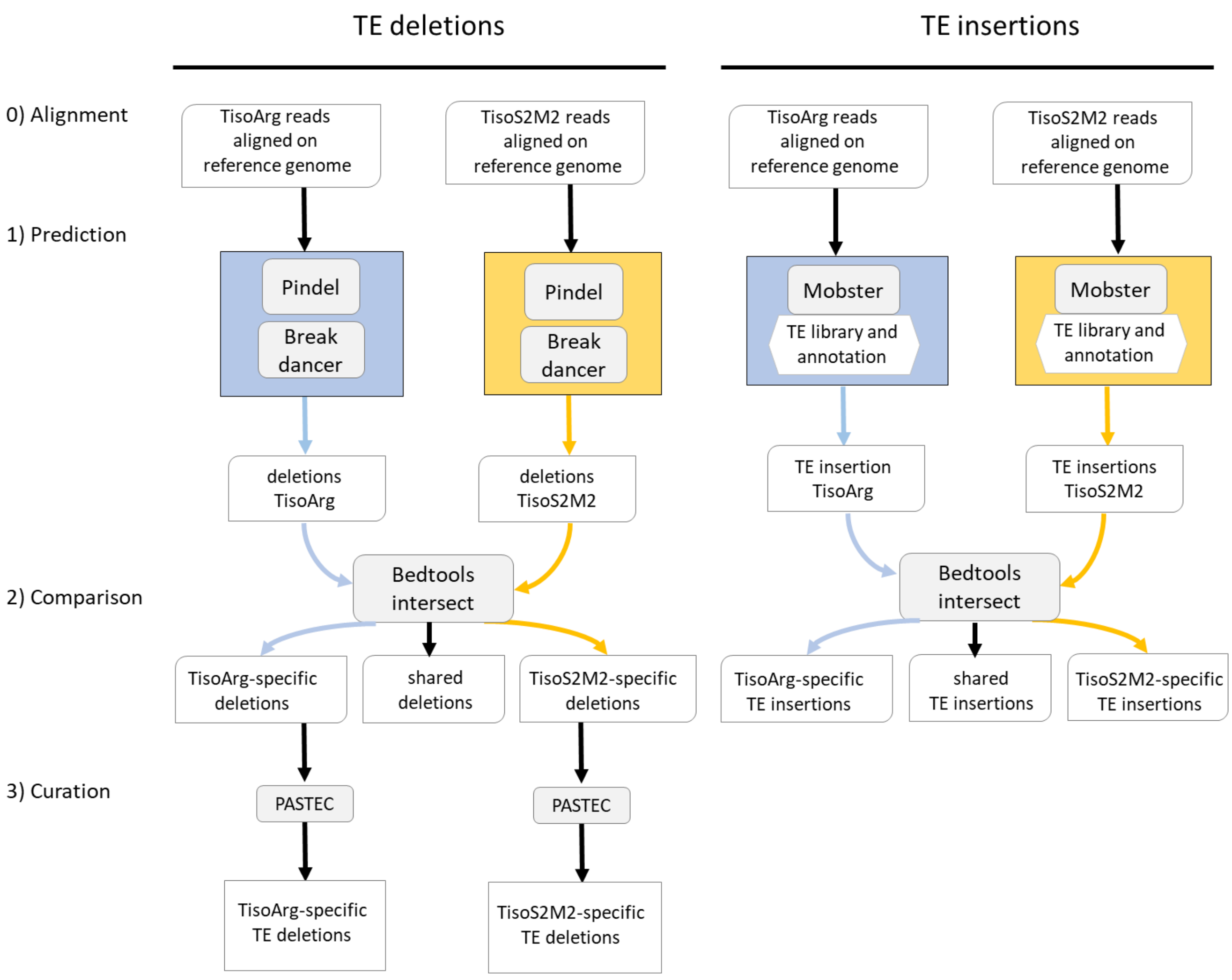

Data S1: Pipeline used to predict specific TE indels in TisoArg and TisoS2M2 using Illumina sequencing data.

0) Alignment step:

The Illumina paired-end reads of each strain (TisoArg and TisoS2M2) were independently aligned on the reference genome.

1) Prediction step:

For each strain, the alignments were submitted to Pindel and Breakdancer to predict deletion events and to Mobster to predict TE insertion events. Only 0/1 and 1/1 predictions were analyzed.

2) Comparison step:

The location of the predicted deletions and TE insertions were extracted. For deletion events: a +/- 1,000 window of location was generated for each deletion event detected by Pindel and

Breakdancer. The windows of location were generated by adding -1,000 bp to the “start” location and +1,000 bp to the “end“ location of the deletion event.

For insertion events: windows of insertion region (“start“ and “end“) were generated by adding + or -1,000 bp to the TE insertion location predicted by Mobster. For each indel prediction, the window of locations obtained for the native TisoArg and domesticated strain TisoS2M2 were compared to detect the shared events. Only the deletion and insertion events of TisoArg and TisoS2M2 which did not overlap were considered specific.

3) Curation step:

To prevent false positives, several filters were applied.

For TE deletions: predicted deletions with a length inferior to 150 bp and superior to 15,000 bp were discarded. The deletion sequences were extracted and submitted to PASTEC to recognize TE indels.

For TE insertion: To prevent false positive detections of TE insertions (such as the prediction of TE sequences already present in the reference genome assembly), a window of +/-100 bp was manually generated for each Mobster prediction. Bedtools’ intersect was used to find Mobster predictions that overlapped TE annotations, and overlapping predictions were discarded. Only TE insertion events predicted by Mobster that occurred with an allele frequency of at least 10% and supported by at least 10 reads were kept as candidates.
