# Supplementary material for: Phenotype stability and dynamics of transposable elements in a strain of the microalga Tisochrysis lutea with improved lipid traits
Source: PLoS One. 2023 Apr 27;18(4):e0284656. doi: 10.1371/journal.pone.0284656 (PMC10138859; doi:10.1371/journal.pone.0284656)
Supplement: S5 Data — (PDF) [file pone.0284656.s010.pdf]

Data S5

TisoS2M2_Pindel_46 T_lutea_GenomeV2.4_Contig_093 1613198

CCAGTGATGGGACGTCAGGTATCAGGTCATCAGGTTTCGCCAACCTGATACCTGATATCAGACCTGATATCAGCCT
GATATAAGGTAGTATAATCTTAGTACGTACGTTATAATGTCAGTATGTAACACTTCTAACTTATGACAACAATCAAA
ATCTAACCGCACATCAGCCGACTTACGAGTACTAGTATACGTACATAGAAATGGCCTCAATTACCGCGCACGACTT
TCGCGCGGCCGGATCTCCCCGACGGCAGACCACTCGAAAGCATTGCCCTCGAGTCCGTCCGCCTCCACCGTTCCCC
AGACTGTGGCCGTGGCCAGGGCGCCGTGCGGCATCGCCAACTCCTGCGCCATTTGACTTGGGGAGGGAGGGAGG
GAGCGGTCTAGGAGCGGGCAGGGGAGGGGAGGGAGGGGAGGGAAGAAGAGAGGGAAGGAGCGAGCGAGCG
AGCGAGCGAGTGATCAAGGCAATGAGGGAGTAAGCGAGGGAGCGAGAGAGTGAGCAGGCGGCTAGGGAGACA
GTGAGACGAGTGGTGAGTGGGACGAGGGATGCGAGAGAGGCGGGGCAGGCGCGGGCGACGAGGGGGTGAGG
GAGGTGAAGGAGGGAGGGAGGGAACGGGCAAGGGACGGAGGGAGGCAAGGGAGGGAGGAGCGGGAGGAAG
GAGAGAGGGAGCGAGCCGTGACCGGGACTCTCCGCGCGCCTGCTGCGTCCTCTCGTGCGTGCGTACAGAGTTTTG
TTTTTGTCCGTCGGTATGAGTTTTGTTTTTGCCCCCTATACCTGATACCTGATATCAGGTCGTCAGCTCCAGAGGAC
CTGTTATCATGGTATCAGGACCTGATATCAGCCTGACGTCCCATCACTG

Predicted sequence by Pindel of the deletion event of 862 bp located at the contig 93 of T. lutea. The full sequence corresponding to a potential non-autonomous TE belonging to the DNA transposons family named Ace. The sequences of the terminal TIRs are highlighted in yellow and the internal degenerated TE sequence in gray.

TisoS2M2_Pindel_43 T_lutea_GenomeV2.4_Contig_142 46892

GTAGGGCGGTCAAACACTGTCCTAACTTCTAAGGAGCAGCCGGTCCACGGCCGCGAAGGCGGCGGCGGGCACCG
CCGGCGCACCTCCGACCGCCGCCGCTGCCTCCCCACGCAGTAGGCGGCCCACGCGTGCACAAGGAGTAGCACGGT
CCCATCGCGCCGCCCGTGTAGGGGCAGCAGCACAGCGCGCGCAGCAGCAGGTGCAGCGGTGCAGCCGCGCAAG
CTCCAAGTTGGCCATGGTGCCCGTGCCGAAGAGCAACTCCCCACTTGCCACTTGGCCGTGCAGCTCCTCGTGCGCG
CGCACGCGCGGCACGTAGCATTCAGTGGGTGCGTGAGTGCGTGGGTGCGTGGGTGCACAGTAGTGCACACAGCA
CCGGCAGTGTGGTGTTAACCCGCTGCTGGTACCAACACTTGCAGATTGAGGTGTTAACGCATGTGCCACACCTAGC
CAATGGAGGTGTTAACGCAAAGTGGGTCAACACCGGGTAAACGTGAACCCATACGCCCTGATCTCAGTTTTGGAA
CGGAGGTAGTATTAGAGTCTACGTTTGTATCCCGCGGTAAGCGACAACCATAACTACTAGGGCGGTCAAACAGGG
GTTTTTTAACTCTTAGTAAGGGTTGGGCTGCATTTTTTGACCGTGGCGCGGTGCCCAATAAAAAAGCTCCGGGGGG
GGCTACCTGACGCTACGAAAATCTTCGCCAAGAAATGGTGCGATCCGCCCCCCCTCTCTGGCTCGGCGGATGGGT
GATTTCGACGAGGTATCAGGGCGCGGCCTGTCCGATTGGCGTGACCATGCGGGTGGGACCTGTGTATTTGGTATC
TGGCGGGGTCTCCCATCCTGATGCATCGTCGAAAACGGGCACGCAGCCCGCCCGGTCTCGACTTGAAGTTGAGCT
TTTCAAGCTTTTGCGATTTTAGCGTCTTCTGTCTTTTTTTGATTACGTTTTGTTGTCGGGGCCCAAGGGGGGGGTCC
GGGTCCTAATGGACCCCGGATATGTGCTAGTTGGGTGTATTGGGAACTTTCTACCGTGAAAAATCCTCAAATTTCC
TCAATTCTGAGATGGTCATTTTTTCACTCCTTATAAAAAAACACTGTTTGACCGCCCTA

Predicted sequence by Pindel of the deletion event of 1,100 bp located at the contig 142 of T. lutea. A part of the predicted indel corresponds to a potential non-autonomous TE of 531 bp belonging to a new DNA transposons family that we named Shanks. The sequences of the terminal TIRs are highlighted in yellow and the internal degenerated TE sequence in gray.
