# Supplementary material for: Phenotype stability and dynamics of transposable elements in a strain of the microalga Tisochrysis lutea with improved lipid traits
Source: PLoS One. 2023 Apr 27;18(4):e0284656. doi: 10.1371/journal.pone.0284656 (PMC10138859; doi:10.1371/journal.pone.0284656)
Supplement: S7 Data — (PDF) [file pone.0284656.s012.pdf]

Data S7

# Deletion event of the hAT/Ace at Contig 93 showing heterozygosity

## T_lutea_GenomeV2.4_Contig_093 1612999 1614260

### Nanopore reads with the TE sequence

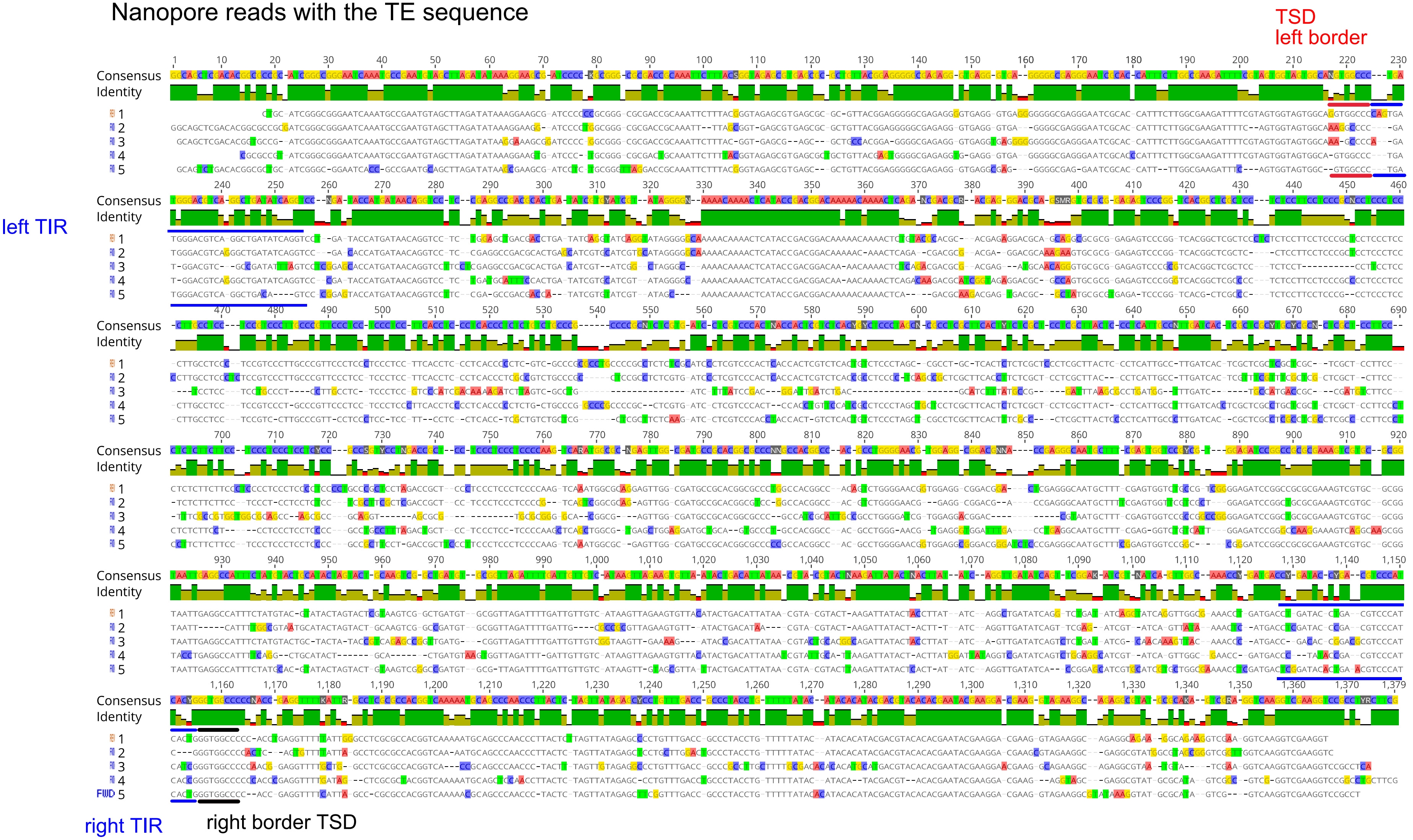


1: ref sequence T_lutea_GenomeV2.4_Contig_093:1612999-1614260
2: read id 75cb6a77-bfca-4fb2-aeed-032dddbf6c7a
3: read id 482b8ee8-9bf9-4557-ad92-d861c6b8e088
4: read id 9554315b-5e7f-4255-9e2b-6d765611df4c
5: read id e14ae163-aa50-4444-acbf-173e90decc26

Nanopore reads without the TE sequence supporting a deletion event

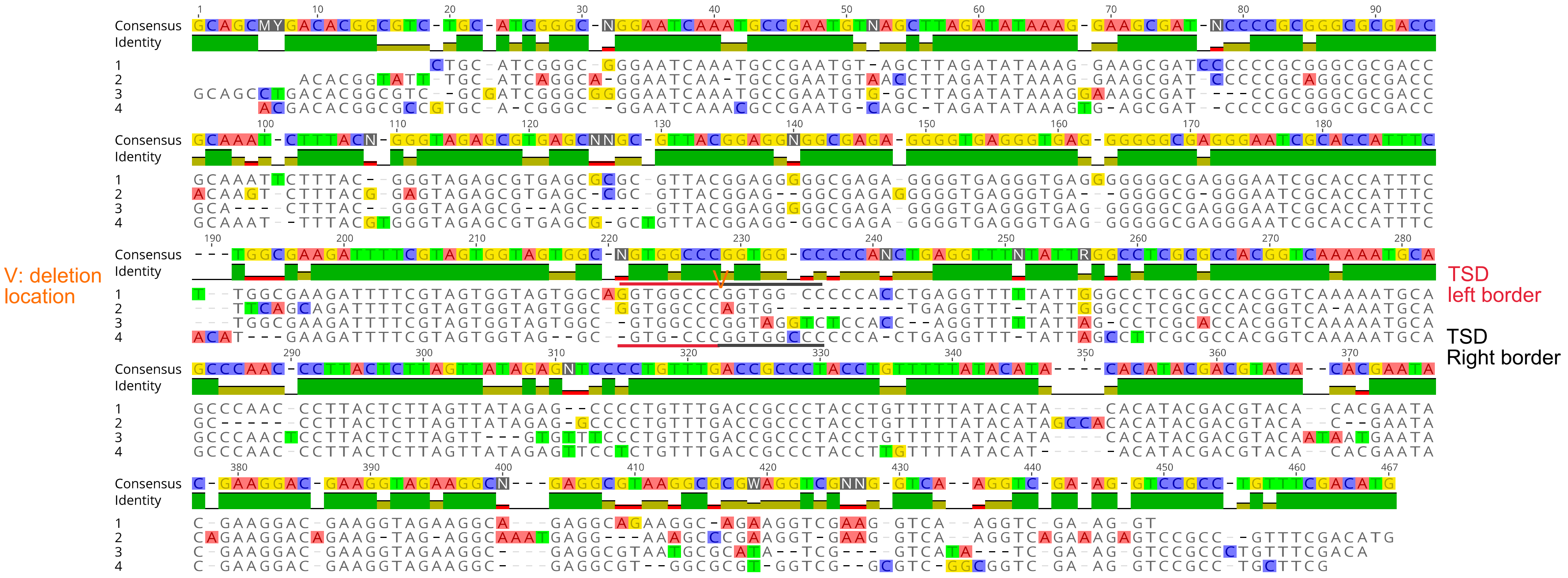


1: ref seq T_lutea_GenomeV2.4_Contig_093:1612999-1614260 without TE seq (manually removed)
2: read id f866e552-f372-463d-86e8-33ce306f3e71 (no clear TSD right border)
3: read id 3899425a-9b6e-406e-a9b1-e54bb8e42171
4: read id 1093223c-c22e-4bec-83ab-d534e179b907
